# Berberine Sensitizes Human Hepatoma Cells to Regorafenib via Modulating Expression of Circular RNAs

**DOI:** 10.3389/fphar.2021.632201

**Published:** 2021-06-17

**Authors:** Kunyuan Wang, Ganxiang Yu, Jiaen Lin, Zhilei Wang, Qianting Lu, Chengxin Gu, Tao Yang, Shiming Liu, Hui Yang

**Affiliations:** ^1^ Department of Gastroenterology, The Second Affiliated Hospital of Guangzhou Medical University, Guangzhou, China; ^2^ Guangzhou Institute of Cardiovascular Disease, The Second Affiliated Hospital of Guangzhou Medical University, Guangzhou, China

**Keywords:** berberine, regorafenib, hepatocellular carcinoma, proliferation, apoptosis, circular RNAs

## Abstract

Regorafenib resistance is a key limiting factor in the treatment of advanced hepatocellular carcinoma (HCC). Increasing evidence has demonstrated that Berberine (BBR) can synergistically enhance the therapeutic effect of various chemotherapeutic agents. However, the contribution of BBR on regorafenib therapy remains unclear. The purpose of this study was to explore the combined treatment effect of berberine and regorafenib in HCC. We found that BBR enhanced the cytotoxicity of regorafenib in HCC cells. Compared with regorafenib alone, the combined treatment of BBR and regorafenib significantly inhibited the proliferation of HCC cells and induced cellular apoptosis. Meanwhile, the combined treatment group with BBR (10mg/kg/day) and regorafenib (5mg/kg/day) had a dramatic inhibitory effect on the growth of HCC xenograft tumors in nude mice. The increased apoptosis of xenograft tumors was seen in the combined treatment group. Moreover, a comprehensive circular RNA sequencing was performed to identify differentially expressed circRNAs in HCC cells after exposure to 100µM BBR and 5µM regorafenib. The volcano plot and scatter plot analyses revealed that there were 58 up-regulated and 19 down-regulated differentially expressed circRNAs between the combination treatment and control groups. Among them, the expression of hsa_circ_0032029 and hsa_circ_0008928 were up-regulated in HCC cells after treatment with 100µM BBR and 5µM regorafenib. Taken together, this study demonstrated that BBR enhanced the anti-HCC effect of regorafenib both *in vitro* and *in vivo*. The synergistic anti-tumor effect of BBR and regorafenib might be related to the up-regulation of hsa_circ_0032029 and hsa_circ_0008928 in HCC cells.

## Introduction

Hepatocellular carcinoma (HCC) is a major health problem with increasing incidence and mortality (Bray et al., 2018; Forner et al., 2018). Although the treatment of HCC has improved over the past decade, the therapeutic options for patients with advanced HCC are very limited (Vogel and Saborowski, 2020). Regorafenib, an oral multi-kinase inhibitor, has become a therapeutic agent which significantly improves overall survival after the treatment failure of sorafenib, and receives the approval as a second line treatment for advanced HCC in 2017 (Bruix et al., 2017; Duffy and Greten, 2017). However, regorafenib also faces the risk of drug resistance and subsequent progression of HCC after treatment (Wang et al., 2019). Therefore, it is needed to find novel agents that can increase the sensitivity of HCC cells to regorafenib treatment.

Chinese medicine has been used worldwide as a supplement and alternative medicine for the treatment of cancers. The experimental studies and clinical trials have shown that Berberine (BBR) can exert anti-cancer activity by inhibiting cellular proliferation and inducing cell apoptosis in various cancers (Fondevila et al., 2019; Lagoa et al., 2020). Recently, a randomized double-blinded, placebo-controlled trial shows that BBR reduces the risk of colorectal adenoma and recurrence of polypoid lesion in patients after polypectomy (Chen et al., 2020). By using Janus nanocarrier containing doxorubicin (DOX) and BBR simultaneously, BBR can drastically enhance the anti-tumor activity of DOX and suppress HCC recurrence (Zhang et al., 2019). In addition, our previous study has proved that BBR makes HCC cells sensitive to sorafenib (Huang et al., 2018). However, it is unclear whether BBR can enhance the anti-HCC activity of regorafenib.

Circular RNAs (circRNAs) belong to the endogenous non-coding RNAs with a closed loop structure, which have multiple biological functions in regulating the occurrence and progression of cancer (Liu et al., 2019). Studies have shown that circRNA is involved in regulating the resistance of chemotherapeutic drugs (Abu et al., 2019; Hua et al., 2019). In the present study, we aimed to investigate the contribution of BBR to the anti-HCC activity of regorafenib and its underlying mechanism. Our data demonstrated that BBR could synergistically enhanced the anti-HCC effect of regorafenib both *in vitro* and *in vivo*. The expression of hsa_circ_0032029 and hsa_circ_0008928 were up-regulated in HCC cells after treatment with 100µM BBR and 5µM regorafenib. These findings suggest that hsa_circ_0032029 and hsa_circ_0008928 may be associated with the synergistic anti-tumor effect of BBR and regorafenib in HCC cells.

## Materials and Methods

### Cell Culture and Reagents

Human HCC cell lines Hep3B and SMMC-7721 were obtained from the Cell Bank of Type Culture Collection (Chinese Academy of Sciences, Shanghai, China) and the American Type Culture Collection (Manassas, VA, USA), respectively. HCC cells were cultivated at 37°C in a 5% CO_2_ incubator with the high-glucose Dulbecco's modified Eagle's medium (Thermo Fisher Scientific, Inc., Waltham, MA, United States), supplemented with 10% (V/V) fetal bovine serum. Regorafenib and BBR were obtained from Selleck Chemicals (Houston, TX, United States; cat. no. S1178 and S2271).

### Cell Viability Assay

HCC cells were seeded into a 96-well plate with 5,000 cells per well. After overnight incubation, HCC cells were treated with the indicated concentrations of regorafenib, BBR or their combination for 24h or 48h. The viability of HCC was examined by MTS Assay (Promega, United States; cat. no. G3588) and Synergy H1/Epoch microplate reader (BioTek Instruments, Inc., Winooski, VT, United States). The cell survival rate was calculated as follows: viability (%) = (average OD value of drug-treated sample/average OD value of control sample) × 100%. The drug concentration (IC50) that inhibited cell growth by 50% was calculated via Probit Regression.

### Combined Effect Evaluation

The interaction between regorafenib and BBR was quantified by Com-puSyn software (ComboSyn, Inc., Paramus, NJ, http://www.combosyn.com/feature.html) and determined by the combination index (CI). CI value < 1, = 1 and >1 represented synergistic, additive and antagonistic effects, respectively.

### EdU Incorporation Assay and Apoptosis Assay

The Cell-Light^TM^ EdU ApolloR567 *In Vitro* Imaging Kit (RiboBio, Guangzhou, China) was used to detect the level of cell proliferation. The Annexin V-FITC/PI Apoptosis Detection Kit (Thermo Fisher, USA) and DeadEnd^TM^ Fluorometric Tunel System assay (Promega, cat. no. G3250; United States) were used to measure cellular apoptotic level. The EVOS FL High Content Imaging System (Invitrogen, Carlsbad, CA, United States) was performed to obtain and analyze images.

### Western Blot Assay

The extraction of total protein lysate and SDS-PAGE were performed as previously described (Huang et al., 2018). After protein extracts were transferred onto methanol-activated polyvinylidene fluoride (PVDF) membranes in 1.5h at 90V condition, the PVDF membranes were blocked with 5% non-fat milk for 1h at 25°C. The membranes were probed with primary antibodies at 4°C for 12h, including anti-poly (ADP-ribose) polymerase (PARP; 1:1,000; cat. no. 9532; Cell Signaling Technology, Inc.), anti-BCL-2 (1:1,000; cat. no. 2870; Cell Signaling Technology, Inc.), and anti-GAPDH (1:1,000; cat. no. 2118; Cell Signaling Technology, Inc.). The membranes were subsequently incubated with horseradish peroxidase-conjugated anti-rabbit or anti-mouse IgG secondary antibody (1:5,000; cat. no. 7074 or 7,076; Cell Signaling Technology, Inc.) for 1h at 25°C. Then, the protein bands were detected by enhanced chemiluminescence (SuperSignal West Pico Chemiluminescent Substrate; Pierce; Thermo Fisher Scientific, Inc.). ImageJ software (National Institues of Health, Bethesda, MD, United States) was used to scanned and analyzed the protein bands.

### *In vivo* Tumor Assays

Male BALB/c nude mice at 4 weeks old were subcutaneously injected with 1×10^7^ SMMC-7721 cells in the underarm region. When tumor size reached 5 × 5mm^2^, mice were randomly divided into four groups (n = 5 in each group). Regorafenib (5mg/kg/day, 5 days per week) and BBR (10mg/kg/day, 2 days per week) were administered by gavage and intraperitoneal injection, respectively. The tumor volume of nude mice was measured by a caliper and calculated according to the formula (width^2^ × length)/2. *In vivo* experiments were performed according to the guidelines for the use of laboratory animals that was approved by the Second Affiliated Hospital of Guangzhou Medical University.

### Circular RNA Sequencing, Differential Expression Analysis and Target miRNA Prediction

Trizol reagent (Invitrogen, Carlsbad, CA, United States) was used to extract the total RNAs from SMMC-7721 cells cultured in DMSO, regorafenib, BBR or their combination. The RNA-Seq libraries were constructed according to Illumina standard protocols and sequenced with Illumina HiSeq 3,000 through Genergy Biotechnology Co., Ltd (Shanghai, China). CircRNAs expression levels were quantified by the number of reads spanning back-spliced junctions (circular reads). CircRNAs expression was expressed as BSRP (back-spliced reads per million mapped reads), which circular reads was normalized to per million mapped reads. The DESEQ software package was used to identify differentially expressed circRNAs in the two groups (DMSO and the combination of regorafenib and BBR) with *t* test *p*-value < 0.05 and fold change >2. The top 77 expressed circRNAs were log2 transformed, gene mean centered and visualized as heatmaps using the Multi Experiment Viewer. Differentially expressed circRNAs were used to predict the potential binding sites of miRNAs by miRanda with threshold parameters as follows: single-residue-pair match scores >150, ΔG < −20kcal/mol and demand strict 5’ seed pairing.

### Real-Time PCR Validation

To detect the expression of circRNAs observed by high-throughput sequencing, five circRNAs were chosen for real-time PCR. Total RNA was extracted from HCC cell samples from DMSO, regorafenib, BBR and combination treatment groups using Trizol reagent. CircRNA levels were quantified by using the Prime Script RT Reagent Kit (TaKaRa, Dalian, China) and SYBR Premix Ex Taq (TaKaRa, Dalian, China). Then, Real-time PCR was performed on ABI Prism 7,300 real-time PCR system (Applied Biosystems, Foster City, CA, United States). Relative quantification was calculated based on the comparative CT method. GAPDH was used as an internal control to normalize the data. Primer sequences are shown in Supplementary Table S1 in Supporting Information.

### Statistical Analysis

Statistical analyses were conducted by SPSS version 16.0 software (SPSS Inc. Chicago, IL, United States). The quantitative data was shown as means ± SD from three independent experiments. Statistical analysis was performed using Student’s *t*-test or one-way ANOVA. A value of *p* < 0.05 indicated a significant difference.

## Results

### BBR Synergistically Enhances the anti-HCC Effect of Regorafenib *in vitro*

**FIGURE 1:**
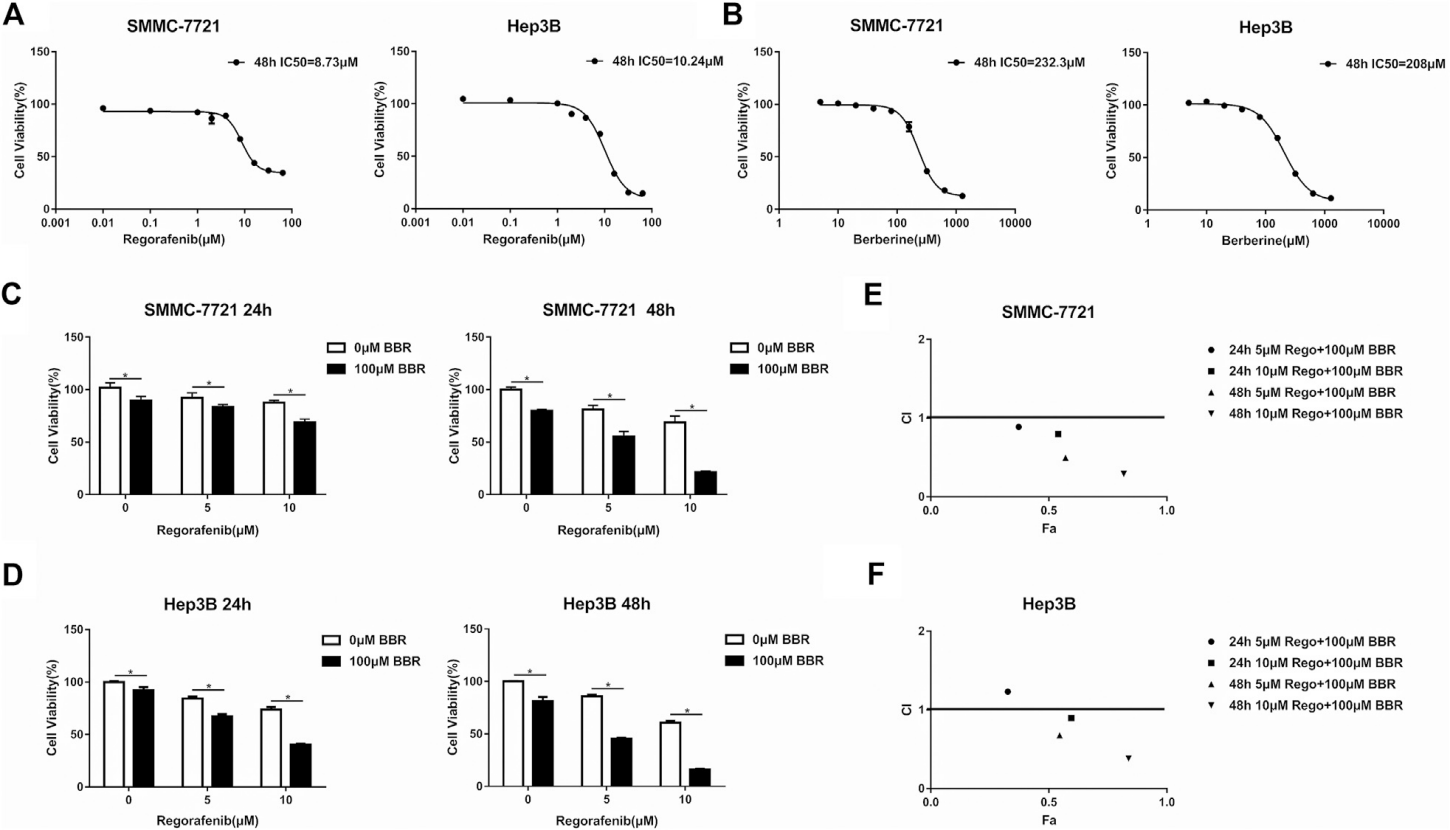
BBR promoted the anti-proliferative effect of regorafenib on HCC cells *in vitro*. **(A,B)** After HCC cells were treated with different concentrations of regorafenib (0, 0.01, 0.1, 1, 2, 4, 8, 16, 32 and 64μM) and BBR (0, 5, 10, 20, 40, 80, 160, 320, 640 and 1280μM) for 48h, MTS assay was used to detect cell viability. **(C,D)** HCC cells were treated with regorafenib (5, 10μM), BBR (100μM) or the combination of regorafenib and BBR for 24h or 48h. MTS assay was used to determine cell viability. **(E,F)** The combination index of each combined treatment was calculated using CompuSyn software. Points below the dotted line indicated synergy (CI values <1). *p < 0.05.

To explore the anti-HCC effect of regorafenib and BBR, the cellular viability was evaluated by MTS assay after HCC cells were treated with regorafenib or BBR at different concentrations. The results showed that regorafenib or BBR alone could inhibit the proliferation of HCC cells in a dose-dependent manner (Figures 1A,B). The IC50 value of regorafenib in SMMC-7721 and Hep3B cells were 8.73 and 10.24µM, respectively (Figure 1A). The IC50 of BBR was 232.3µM in SMMC-7721 cells and 208µM in Hep3B cells (Figure 1B). Moreover, the IC50 of BBR in normal hepatocytes L02 cells was 408.9µM, indicating that BBR at a concentration of 100µM had no obvious inhibitory effect on normal liver cells (Supplementary Figure S1A). Since the trough blood concentration of regorafenib in patients receiving regorafenib 40–160mg/day was 318–9467ng/ml (Taguchi et al., 2020), which was equivalent to 0.612–18.231µM, we first detected the combined effect of 1µM regorafenib and 100µM BBR. The results showed that 100µM BBR had a cytotoxic effect on HCC cells, while 1µM regorafenib had no influence on HCC cell viability (Supplementary Figure S1B). And the CI values of 1µM regorafenib and 100µM BBR combination in SMMC-7721 and hep3B cells were both greater than 1 (Supplementary Figure S1C). Therefore, regorafenib (5 and 10µM) and 100µM BBR were selected for further study. We found that the anti-proliferative effect caused by the combined treatment of regorafenib and BBR was significantly greater than that by a single agent treatment (Figures 1C,D). As shown in Figure 1E, regardless of 24h or 48h treatment, the CI values of different drug combinations in SMMC-7721 cells were all less than 1. In Hep3B cells, except for the combination of 5µM regorafenib and 100µM BBR for 24h, the CI values of other combined groups was less than 1 (Figure 1F). The above results indicate that there is a synergistic interaction between BBR and regorafenib in inhibiting the proliferation of HCC cells.

### Combined Treatment of BBR and Regorafenib Significantly Inhibits the Proliferation of HCC Cells

**FIGURE 2:**
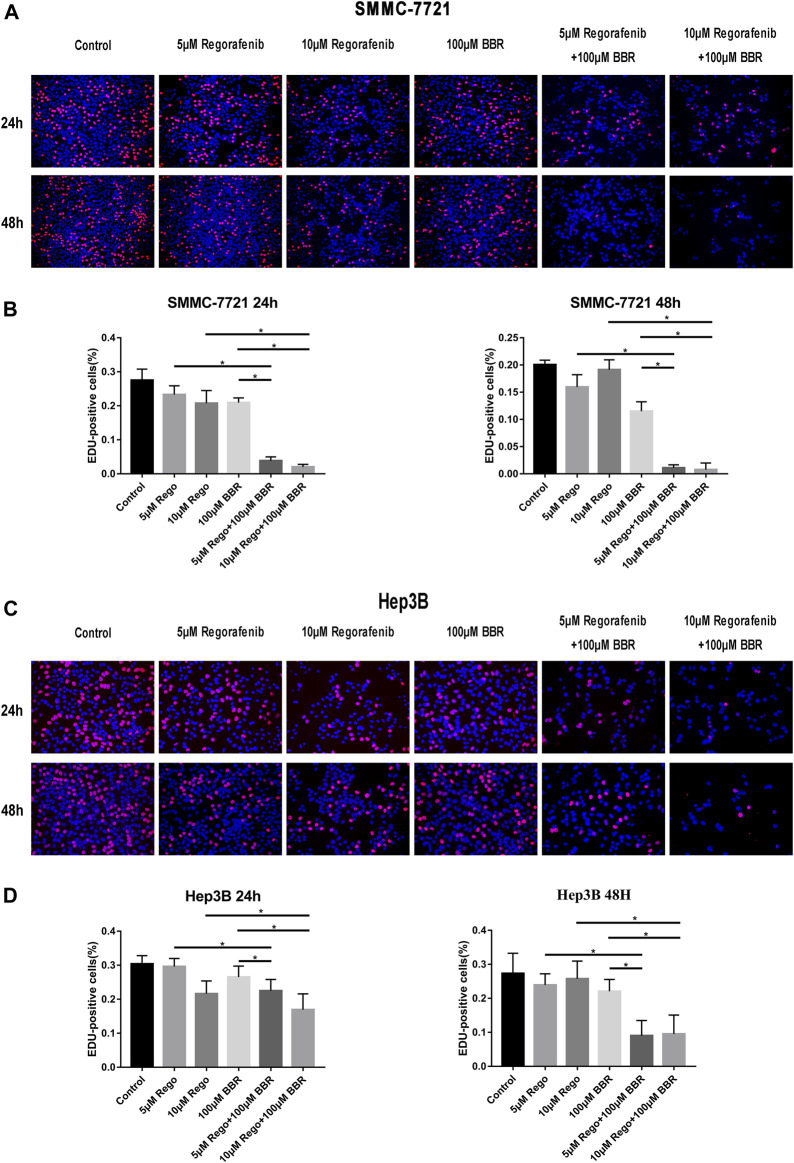
The combination of BBR and regorafenib reduced the proliferative capacity of HCC cells. **(A,B)** The proliferative capacity of SMMC-7721 cells detected by Edu assay after treatment with regorafenib (5, 10µM), BBR (100µM) alone or in combination for 24 or 48h. **(C,D)** The combination of BBR and regorafenib reduced the proliferative capacity of Hep3B cells, as determined by Edu assay (magnification, ×200). *p < 0.05.

Then, we applied EdU assay to detect the anti-proliferative effect of BBR and regorafenib. We observed that compared with regorafenib or BBR alone, the combined therapy of regorafenib and BBR obviously inhibited the proliferation of HCC cells (Figures 2A,B). The combined treatment with 10µM regorafenib and 100µM BBR suppressed HCC cells proliferation more obviously than that with 5µM regorafenib and 100µM BBR. Meanwhile, the group treated with 10µM regorafenib and 100µM BBR for 48h has a stronger proliferation inhibitory effect when compared with other groups (Figures 2C,D). These results demonstrate that a longer drug exposure time will produce a stronger inhibitory effect.

### Co-Treatment of BBR and Regorafenib Induces HCC Cells Apoptosis

**FIGURE 3:**
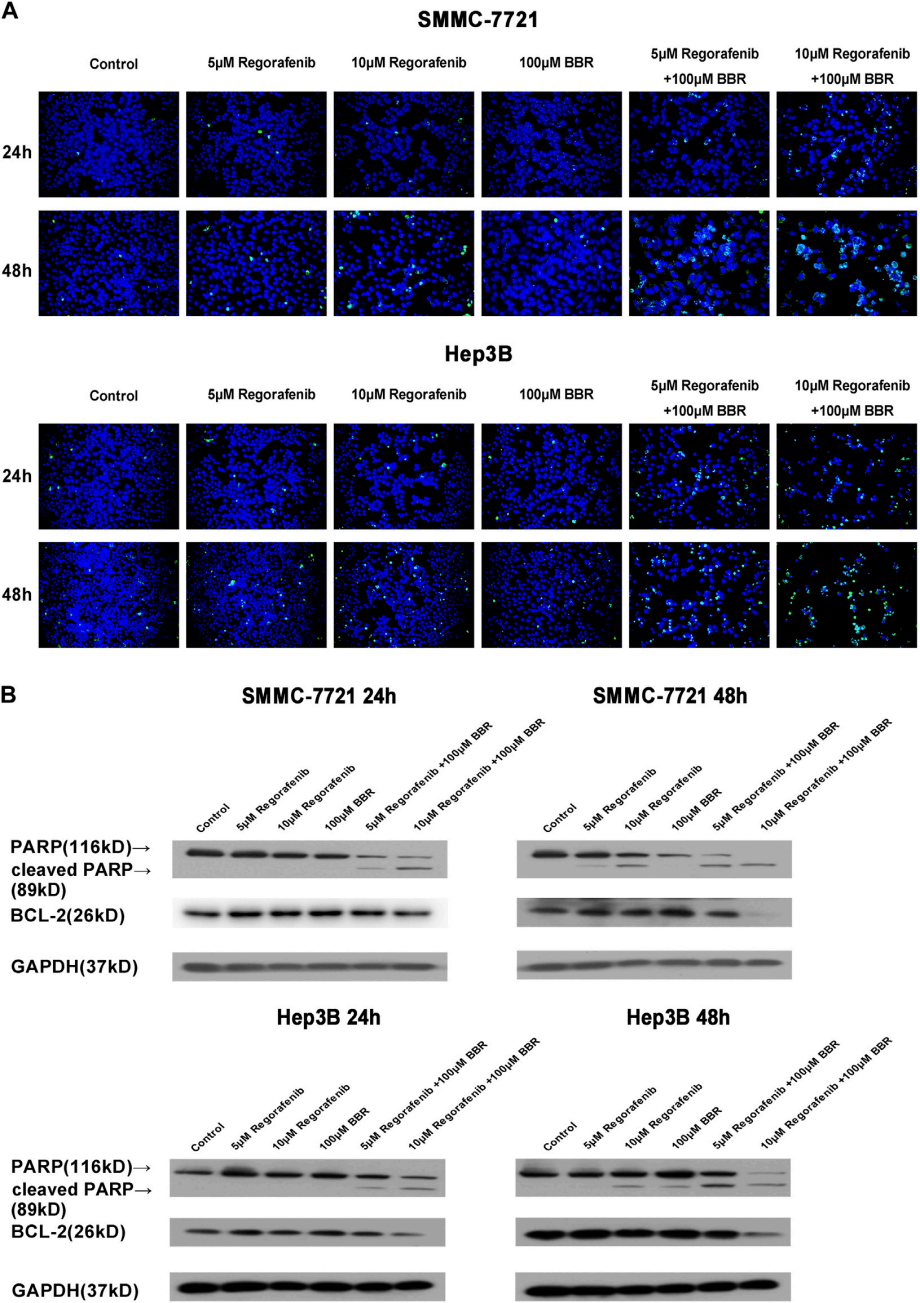
Apoptosis was induced by the combination treatment of BBR and regorafenib in HCC cells. **(A)** HCC cells were treated with regorafenib (5, 10µM), BBR (100µM) alone or in combination for 24 or 48h. The apoptotic cells were examined by TUNEL assay (200 x). **(B)** Western blotting assay measured the expression of apoptosis related proteins. GAPDH was used as a loading control.

To investigate cellular apoptosis induced by BBR and regorafenib, HCC cells were treated with BBR and regorafenib alone or in combination. We found that the combined treatment of BBR and regorafenib significantly induced HCC cells apoptosis when cells were cultured over 48h (Figure 3A). The protein expression of cleaved PARP was up-regulated, while the expression of anti-apoptotic protein BCL-2 was down-regulated in BBR and regorafenib combined treatment group (Figure 3B).

**FIGURE 4:**
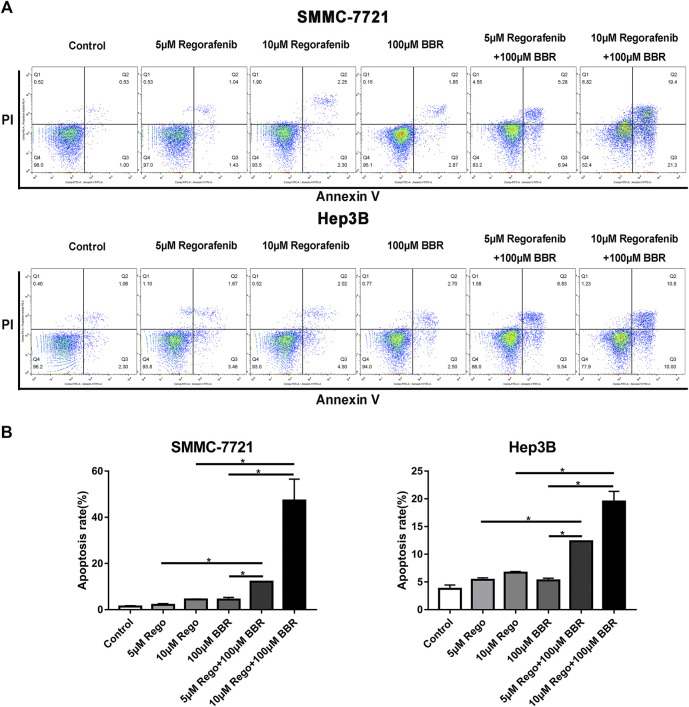
BBR enhanced regorafenib induced apoptosis in HCC cells. **(A)** Flow cytometric assay analyzed the apoptotic rate of SMMC-7721 and Hep3B cells induced by BBR and regorafenib. After HCC cells were treated with BBR (100µM), regorafenib (5, 10µM) or their combination for 48h, they were stained with Annexin V/PI. **(B)** Histogram displayed the apoptotic rates in different groups from three independent experiments. *p < 0.05.

Meanwhile, Annexin V-FITC/PI co-staining was performed to further confirm the apoptosis-inducing effect of the combined treatment. After 48h of exposure to the series of treatments, we observed an increase in the percentage of Annexin V-positive cells in the cells treated with combined 10μM regorafenib and 100μM BBR (Figure 4A). Compared with the control group, the monotherapy or combination treatment group of 5µM regorafenib and 100µM BBR, the fluorescence intensity of Q2 and Q3 regions showed that the number of apoptotic cells was the highest in the combinational treatment group of 10µM regorafenib and 100µM BBR (Figure 4B). These results suggested that the combined treatment of regorafenib and BBR had synergistic effect in inducing cell apoptosis.

### BBR Enhances the Anti-Tumor Effect of Regorafenib *in vivo*

**FIGURE 5:**
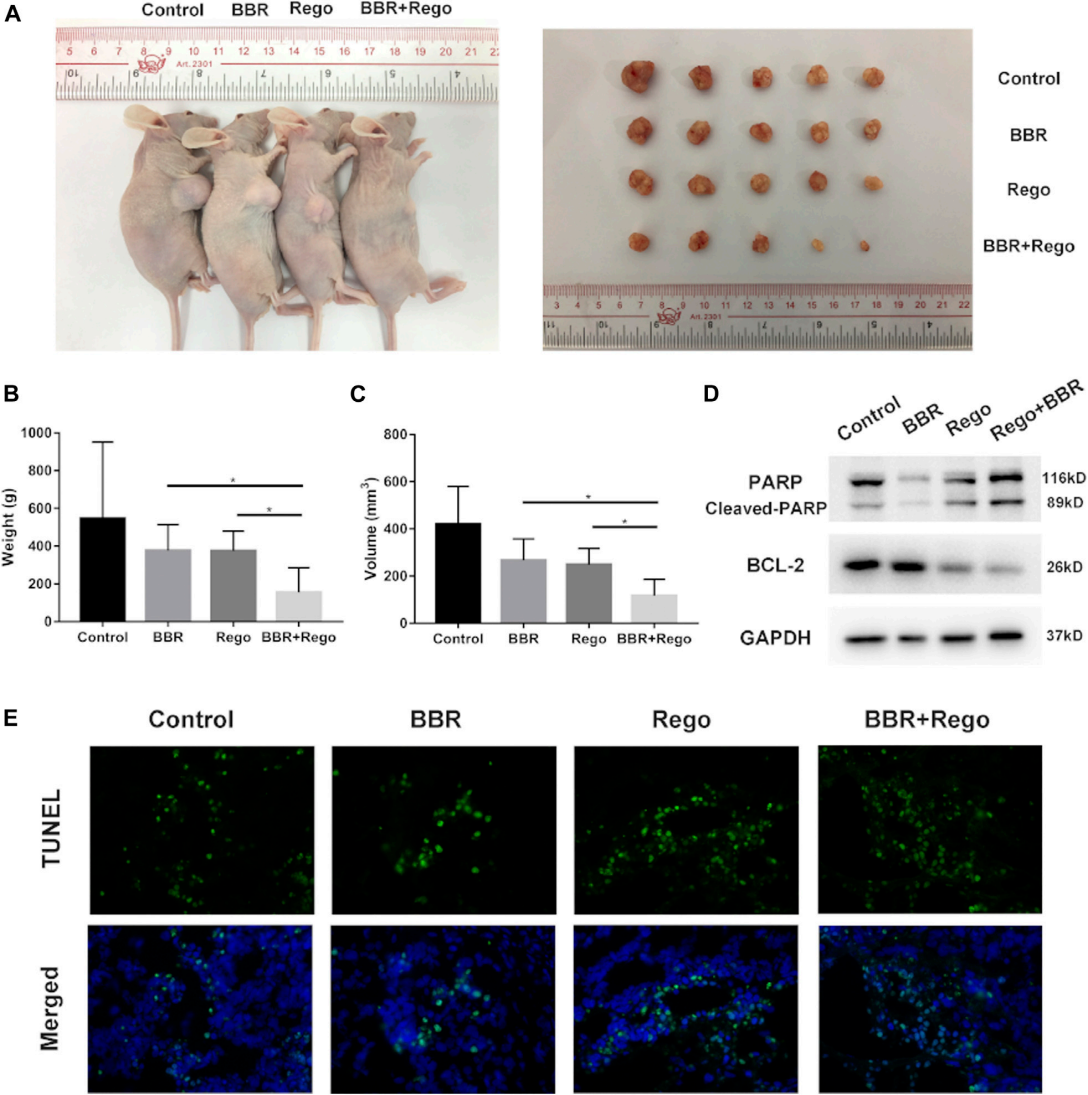
Combined treatment with BBR and regorafenib inhibited the growth of xenograft tumors and induced cell apoptosis in mice. **(A)** Representative images of the subcutaneous xenografts tumors in mice treated with DMSO, BBR, regorafenib and their combination. **(B,C)** The weight and volume of xenograft tumors displayed as mean ± SD in DMSO, BBR, regorafenib and the combined treatment groups. **(D)** Western blotting assay measured the expression of apoptosis related proteins. GAPDH was used as a loading control. **(E)** TUNEL assay was used to evaluate the apoptosis of xenograft tumor tissue. n = 5, *p < 0.05.

Next, we applied a subcutaneous xenograft tumor model to validate the anti-HCC effect of BBR and regorafenib *in vivo*. Tumor xenograft was established by transplanting SMMC-7721 cells into nude mice. BBR (10mg/kg/day) and regorafenib (5mg/kg/day) were administered to tumor-bearing mice alone or in combination. The results showed that the tumor size was smaller in the mice of combined treatment group compared with that in the DMSO group, BBR alone group and regorafenib alone group (Figure 5A). The tumor volume and weight of nude mice were dramatically reduced in the combined treatment group as compared with other groups (*p* < 0.05; Figures 5B,C). Moreover, the protein expression of cleaved PARP was up-regulated in xenograft tumor tissues in the BBR and regorafenib combined treatment group, while the protein expression of BCL-2 was down-regulated (Figure 5D). We also found that, as shown by TUNEL staining, the apoptosis level of xenograft tumors was much higher in the combination treatment group (Figure 5E). These results indicated that the combined treatment of BBR and regorafenib suppressed HCC cells growth and induced apoptosis *in vivo*.

### Expression Profiles of circRNAs in HCC Cells Treated with BBR and Regorafenib

**FIGURE 6:**
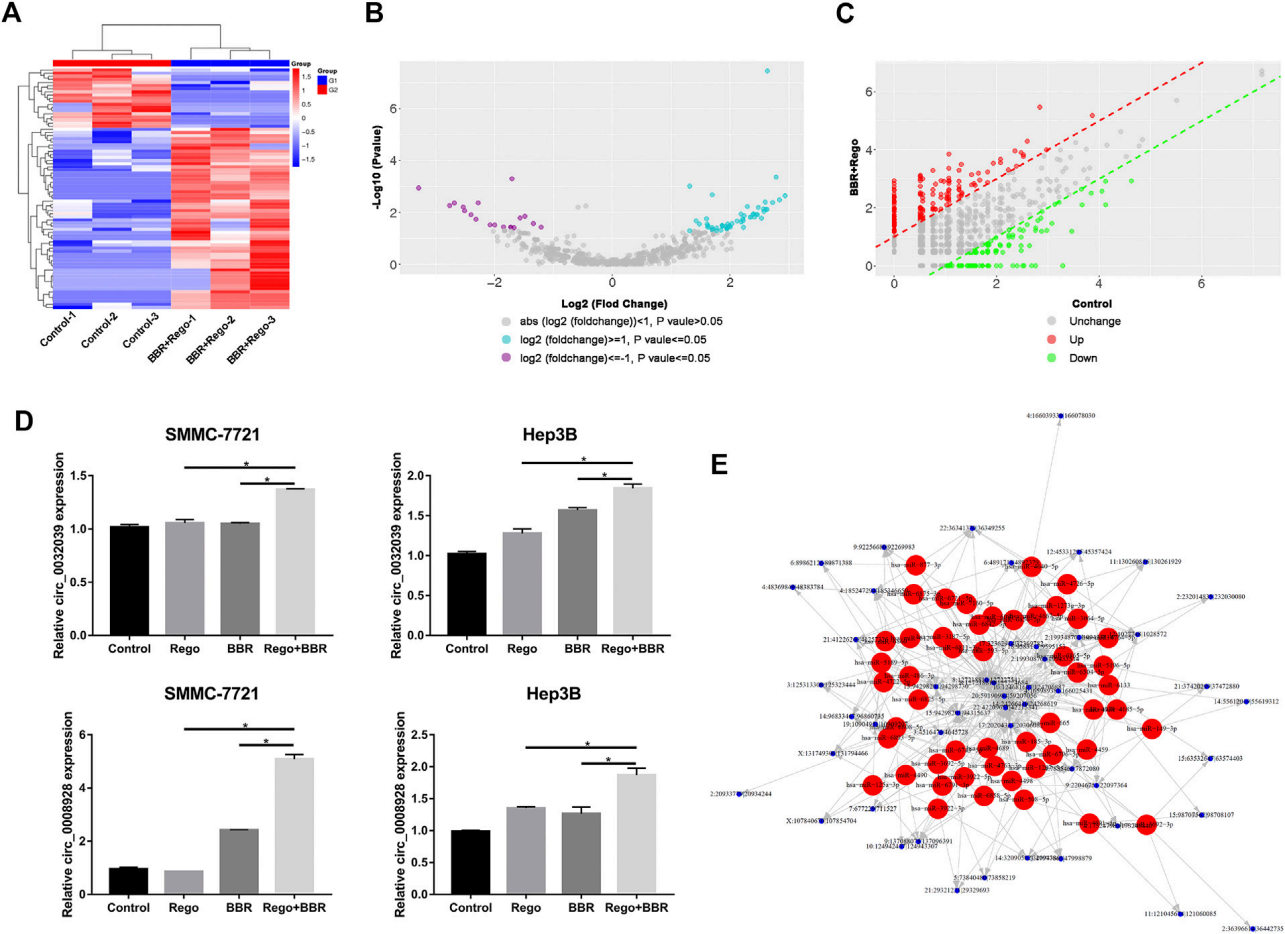
Differential expression of circRNAs in SMMC-7721 cells treated with BBR and regorafenib. **(A)** Different circRNA expression profiles among samples from the RNA sequencing data shown by heat map. Three DMSO samples and three combined samples of BBR and regorafenib were examined. **(B,C)** In the volcano and scatter plots, 58 circRNAs were increased and 19 were decreased in the combination treatment group. **(D)** qRT-PCR performed on the validation of two differentially expressed circRNAs in control, BBR, regorafenib and the combination groups. **(E)** Interaction network of circRNAs and their potential target miRNAs *p < 0.05.

To investigate the potential mechanism of BBR and regorafenib combination, we initiated a comprehensive circular RNA sequencing to identify differentially expressed circRNAs in HCC cells after exposure to 100µM BBR and 5µM regorafenib. DEGseq analysis was applied to search for the differentially expressed circRNAs, and clustered according to their expression profiles (Figure 6A). As presented in Figures 6B,C, volcano plot and scatter plot analyses revealed that there were 58 up-regulated and 19 down-regulated differentially expressed circRNAs between the combination treatment and control groups. To validate the results of circular RNA sequencing, we used qPCR assay to detect the expression level of five circRNAs (hsa_circ_0032029, hsa_circ_0008928, hsa_circ_0001346, hsa_circ_0006702, hsa_circ_0008039) that were up-regulated in HCC cells after treatment with 100µM BBR and 5µM regorafenib. The results showed that the expression of hsa_circ_0032029 and hsa_circ_0008928 were up-regulated (Figure 6D), and there was no change on the expression hsa_circ_0001346, hsa_circ_0006702 and hsa_circ_0008039 (Supplementary Figure S2).

In addition, we predicted the miRNA binding sites for differential expressed circRNAs using miRanda software. According to the *p*-value ranking information, the top 50 target circRNAs that were differentially expressed in the control and combination treatment groups were selected. We then matched them to 50 potential target miRNAs. Finally, an interactive network map was constructed based on these data (Figure 6E). These up-regulated circRNAs will be candidates for further study on the mechanism of the combination treatment with BBR and regorafenib.

## Discussion

Drug resistance and toxicity remain key issues in the treatment of advanced HCC. Although regorafenib treatment can significantly improve the overall survival of patients with HCC, resistance to regorafenib is inevitable (Bangaru et al., 2020). Novel strategies are needed to enhance the efficacy of regorafenib in HCC treatment. In this study, we explore the effect of BBR in sensitizing HCC cells to regorafenib both *in vitro* and *in vivo*.

As a multi-kinase inhibitor, regorafenib plays its anti-tumor role by blocking the activity of multiple protein kinases that involve in cancer cell proliferation, metastasis, angiogenesis and tumor microenvironment (Teufel et al., 2019). Regorafenib alone has been reported to inhibit the proliferation of HCC cells. Similar to previous studies (Refolo et al., 2018; Wang et al., 2020), our data indicated that regorafenib dramatically inhibited the proliferation of HCC cells in a time- and dose-dependent manner. Several preclinical studies have shown that some chemotherapeutic agents can effectively improve the anti-tumor efficacy of regorafenib. Chlorogenic acid can enhance the inhibitory effect of regorafenib in cell growth and motility, and potentiate regorafenib-induced apoptosis in HCC cells (Refolo et al., 2018). B. Wang et al. observed that regorafenib and ginsenoside combined treatment significantly suppressed the proliferation of liver cancer cells (Wang et al., 2020). Researchers found that ramucirumab and GSK1838705A (VEGFR2 and IGF1R inhibitors) increased the sensitivity of HCC cells to the combination of sorafenib and regorafenib at low concentrations (D''Alessandro et al., 2019).

Numerous studies demonstrated the beneficial role of BBR in preventing cancer and enhancing chemotherapeutic effect when combined with BBR in HCC treatment (Liu et al., 2019a). BBR can inhibit HCC cell proliferation *in vitro* and tumor xenografts growth *in vivo* (Chuang et al., 2017; Zhang et al., 2019a). We found that BBR alone suppressed the proliferation of SMMC-7721 and hep3B cells in a concentration-dependent manner in this research. Furthermore, BBR has exhibited the ability to overcome multidrug resistance. BBR sensitized the cells to paclitaxel, and combination of BBR and paclitaxel resulted in potentiation, that inhibited the growth of tumors and elicited apoptosis of cancer cells (Cheng and Ji, 2020). BBR could also sensitize MDA-MB-231 cells to camptothecin, cisplatin and methyl methanesulfonate (Gao et al., 2019). Our study indicated that BBR and regorafenib combined treatment had a remarkable anti-HCC effect via inhibiting cellular proliferation and inducing cell apoptosis both *in vitro* and *in vivo*. Simultaneously, we found this phenomenon was more pronounced in the combinational of 10µM regorafenib and 100µM BBR than in combination of 5µM regorafenib and 100µM BBR alone group, suggesting that the synergic effect of regorafenib and BBR was concentration-dependent. This study further proves that BBR has the potential to improve chemotherapeutic efficacy.

Previous studies have identified various differentially expressed circRNAs in multiple diseases, particularly in human cancers (Liu et al., 2019). Some dysregulated circRNAs are related to chemotherapeutic resistance (Hua et al., 2019). It had been reported that circRNA-104797 was up-regulated in sorafenib-resistant HCC cells, and the depletion of circRNA-104797 increased the sensitivity of sorafenib in HCC cells (Xu et al., 2020). Su et al. identified circELP3, a hypoxia-elevated circular RNA, contributed to bladder cancer progression and cisplatin resistance (Su et al., 2019). CircPVT1 was up-regulated in chemo-resistant osteosarcoma cells, and knockdown of circPVT1 could impair the resistance of osteosarcoma cells to doxorubicin and cisplatin (Kun-Peng et al., 2018). Additionally, Chen et al. found that circ-0003418 exerted an anti-tumor role in HCC and increased the sensitivity of HCC cells to cisplatin (Chen et al., 2019). RNA sequencing is a technology that can be applied to detect potential biomarkers or candidate therapeutic targets in various diseases. In this study, we performed circular RNA sequencing to identify differentially expressed circRNAs in HCC cells after cells were treated with 100µM BBR and 5µM regorafenib. The results demonstrated that the expression of hsa_circ_0032029 and hsa_circ_0008928 were up-regulated after cells were treated with 100µM BBR and 5µM regorafenib, suggesting that these circRNAs may play a role in modulating the anti-tumor activity of the combination of BBR and regorafenib. CircRNAs can regulate tumor cell phenotype by acting as a miRNA sponge, transcription regulator or by protein interaction, that correlate with the subcellular location of circRNAs (Cui et al., 2020). Usually, circRNAs that were predominantly localized in the cytoplasm serve as molecular sponges of miRNAs or proteins, while circRNAs localized in the nucleus play their role by regulating transcription. Currently, it was demonstrated that circARNT2 was localized in the cytoplasm of HCC cells and could regulate cell proliferation, apoptosis and cisplatin resistance in HCC through acting as a sponge for miR-155-5p (Li et al., 2020). CircRNA-104797 regulated the cell-killing ability of sorafenib in HCC via binding to YBX1 in the cytoplasm and preventing the degradation of YBX1 mediated by PRP19 (Xu et al., 2020). Yang et al. found that Circ-CTNNB1 was predominantly localized in the nucleus and promoted cancer progression via DDX3-mediated transactivation of YY1 (Yang et al., 2019). Although circRNAs are generally regarded as non-coding RNAs, several protein-coding circRNAs have been identified (Liu et al., 2020; Wu et al., 2021). The peptides encoded by circRNAs are usually truncated, and their functions are similar to their full-length protein counterparts (Zhou et al., 2020). In the follow-up study, we will detect the sub-cellular localization of hsa_circ_0032029 and hsa_circ_0008928, and investigate how these two circRNAs regulate the synergistic effect of BBR and regorafenib in inhibiting HCC growth.

In summary, our study has demonstrated that the combination of BBR and regorafenib inhibits cellular proliferation and induces apoptosis of HCC cells both *in vitro* and *in vivo*. Both hsa_circ_0032029 and hsa_circ_0008928 could be potential biological targets involved in regulating the synergistic effect of BBR and regorafenib. This research provides evidence for the combinational use of BBR and regorafenib as a novel chemotherapeutic strategy for HCC treatment.

## Data Availability

The datasets presented in this study can be found in online repositories. The names of the repository/repositories and accession number(s) can be found below: NCBI BioProject PRJNA701123.
