# Supplementary figures and images for: Berberine Sensitizes Human Hepatoma Cells to Regorafenib via Modulating Expression of Circular RNAs
Source: Front Pharmacol. 2021 Jun 17;12:632201. doi: 10.3389/fphar.2021.632201 (PMC8248669; doi:10.3389/fphar.2021.632201)

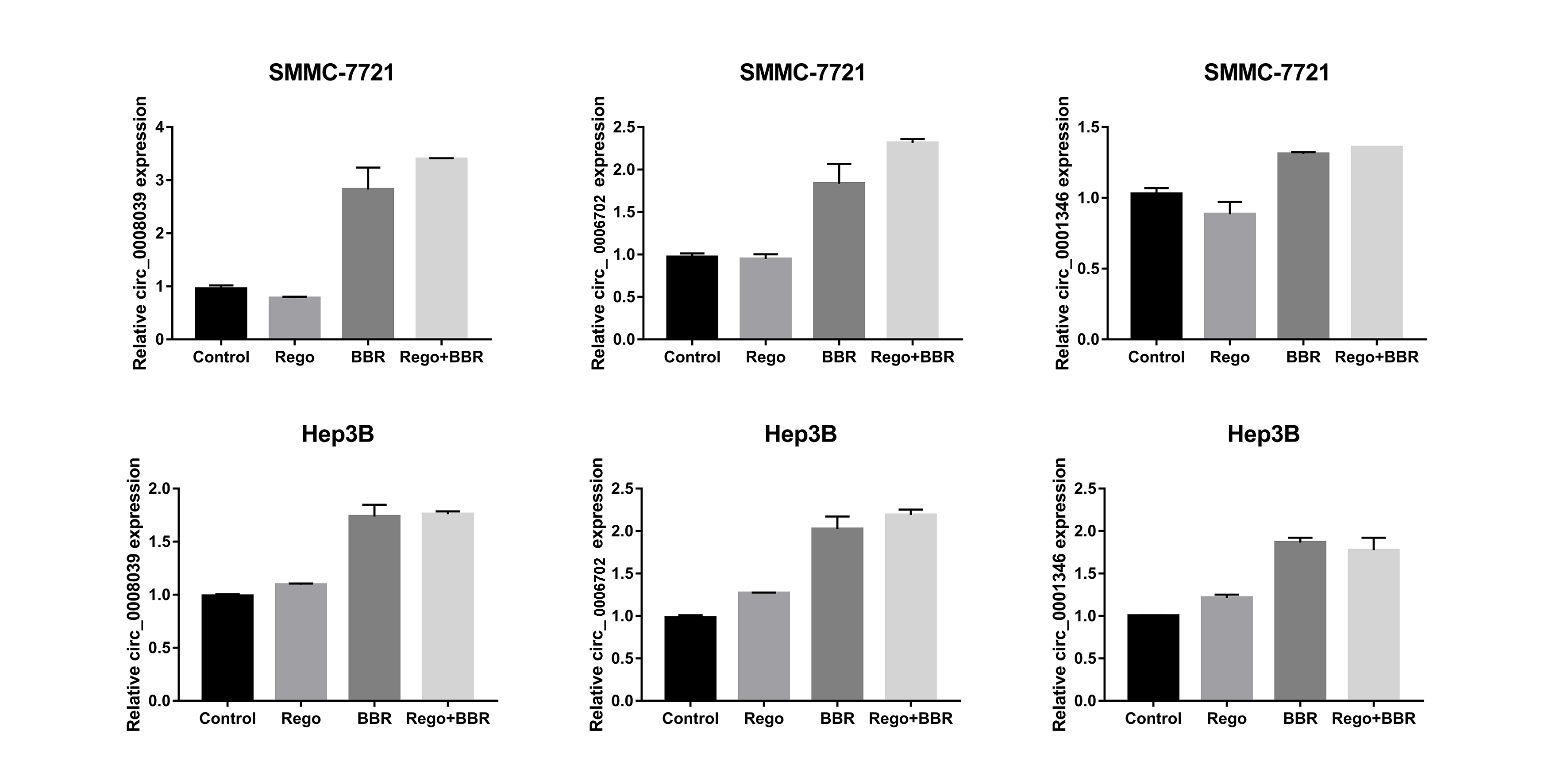

Supplement: Supplementary file 1 [file image1.tif]
